# Psoriasin, a novel anti-*Candida albicans* adhesin

**DOI:** 10.1007/s00109-018-1637-6

**Published:** 2018-05-07

**Authors:** Annelie Brauner, Cathrin Alvendal, Milan Chromek, Konrad H. Stopsack, Sophia Ehrström, Jens M. Schröder, Nina Bohm-Starke

**Affiliations:** 1 Department of Microbiology, Tumor and Cell Biology, Division of Clinical Microbiology, Karolinska Institutet and Karolinska University Hospital, Stockholm, Sweden; 2 Department of Clinical Sciences, Division of Obstetrics and Gynecology, Danderyd Hospital and Karolinska Institutet, Stockholm, Sweden; 3 Department of Clinical Science, Intervention and Technology, Division of Pediatrics, Karolinska Institutet and Karolinska University Hospital, Stockholm, Sweden; 4 Department of Dermatology, Venerology and Allergology, University Hospital Schleswig-Holstein, Kiel, Germany

**Keywords:** *Candida albicans*, Psoriasin, β-Glucan, Adhesion, Immune response

## Abstract

**Abstract:**

*Candida albicans* belongs to the normal microbial flora on epithelial surfaces of humans. However, under certain, still not fully understood conditions, it can become pathogenic and cause a spectrum of diseases, from local infections to life-threatening septicemia. We investigated a panel of antimicrobial proteins and peptides (AMPs), potentially involved in mucosal immunity against this pathogen. Out of six studied AMPs, psoriasin was most up-regulated during a mucosal infection, an acute episode of recurrent *Candida* vulvovaginitis, although candidacidal activity has not been demonstrated. We here show that psoriasin binds to β-glucan, a basic component of the *C. albicans* cell wall, and thereby inhibits adhesion of the pathogen to surfaces and increases IL-8 production by mucosal epithelial cells. In conclusion, we show a novel mechanism of action of psoriasin. By inhibiting *C. albicans* adhesion and by enhancing cytokine production, psoriasin contributes to the immune response against *C. albicans*.

**Key messages:**

- The antimicrobial peptide psoriasin is highly up-regulated during a local mucosal infection, *Candida albicans* vulvovaginitis.
- Psoriasin binds to β-glucan in the *Candida albicans* cell wall and thereby inhibits adhesion of the pathogen.
- Binding of psoriasin to *Candida albicans* induces an immune response by mucosal epithelial cells.

**Electronic supplementary material:**

The online version of this article (10.1007/s00109-018-1637-6) contains supplementary material, which is available to authorized users.

## Introduction

*Candida albicans* is a commensal fungus, colonizing mucosal and cutaneous surfaces of healthy individuals. As an opportunistic pathogen, *C. albicans* can cause a variety of diseases, ranging from trivial local to life-threatening systemic infections. Immunocompromised patients are mostly at risk for severe fungal infections [1]. Indwelling catheters constitute another risk factor, due to the ability of the fungus to adhere and form biofilm on foreign surfaces [2]. Yet, factors of the host-pathogen interactions that lead to asymptomatic carriage, to local infection, or to invasive systemic infection are still not fully understood.

The mucosal epithelium with its normal microbial flora constitutes an important dynamic barrier for invading microorganisms. The epithelial cells produce mucus, anti-adhesins, antimicrobial proteins and peptides (AMPs), chemokines, and cytokines [3–7]. AMPs are mostly cationic, amphipatic, and typically with between 12 and 50 amino acid residues in length [8]. The antimicrobial protein psoriasin (S100A7), however, is larger, not cationic (MW 11,457; pI 6.77) [9] and has highly potent *E. coli-*cidal activity [10]. Unlike most AMPs, it is not acting via membrane effects, but similar to calprotectin [11], in a zinc-dependent fashion [10]. In an approach to explain the resistance of lesional psoriatic skin towards dermatophyte infection, we recently identified the cysteine-reduced form of psoriasin as a fungicidal AMP, which acts via intracellular zinc-depletion and induction of apoptosis-like fungal cell death [12]. Cysteine-reduced psoriasin, but not its oxidized form, is a powerful broad-spectrum fungicide for many dermatophytes and *Aspergillus fumigatus*. Interestingly, however, both the oxidized and the cysteine-reduced forms of psoriasin lack activity towards *C. albicans* [12].

In this study, we sought to investigate the interaction between *C. albicans* and epithelial defense mechanisms, in particular AMPs, during a typical mucosal *C. albicans* infection, vulvovaginal candidiasis. Although lacking a direct antifungal effect, psoriasin was strongly up-regulated during the acute *C. albicans* infection. This AMP supported the mucosal immunity by alternative mechanisms, namely by inhibition of *C. albicans* adhesion and by affecting the immune response.

## Materials and methods

### Participants and samples

The clinical part of the study was approved by the Regional Ethical Review Board in Stockholm. All participants were recruited at the gynecology outpatient clinic at Danderyd Hospital, Stockholm, Sweden, and informed consent was obtained. Recurrent vulvovaginal candidiasis (RVVC) was defined according to Fidel [13], and only cases of primary RVVC were included, i.e., no known predisposing underlying conditions including pregnancy, antibiotic treatment, diabetes, and immunosuppression. Infection with *C. albicans* was confirmed microbiologically; patients infected with other *Candida* spp. or bacterial vaginosis were excluded from the study. None of the patients had received any antibacterial or antifungal treatment at the time of the first sampling. At the first visit, all patients showed typical symptoms of an acute vulvovaginal *C. albicans* infection, and oral antifungal treatment was initiated, consisting of 50 mg/day fluconazole for 1 week and 150 mg/week for 5 weeks. Samples were collected as described below, before and 2–3 weeks after antifungal treatment was completed. A total of 16 women, aged 20–39 years (median 32 years), were included in the study. All patients were culture-negative for *C. albicans* and other urogenital infections at the time of the second visit. Control samples were collected once from 27 healthy female volunteers, aged 19–40 years (median, 25 years). The usage of contraceptives did not differ between patients and control participants (Supplementary Table 1).

### Isolation and identification of *Candida albicans*

For identification of *C. albicans*, vaginal swabs were cultured on CHROMagar Candida and typical colonies were further analyzed by latex surface antigen agglutination (Bichro-Dubli) at the Department of Clinical Microbiology, Karolinska University Hospital, Stockholm, Sweden. Absence of fungal and bacterial growth in the samples from control participants was confirmed according to the same procedure. Isolates were kept frozen at − 80 °C. For experiments, if not stated otherwise, isolates were cultured on Sabouraud agar for 1–2 days at 37 °C and then in yeast peptone dextrose (YPD) medium overnight at 30 °C. This culture was diluted 1:100 in fresh medium and grown for another 3 h at 30 °C to the logarithmic growth phase. Under these conditions, *C. albicans* grew as yeast cells.

### Vaginal lavage

Vaginal lavage was obtained according to a modified method by Valore et al. [14]. Briefly, 5 ml of distilled water was administered intravaginally, and lavage samples were collected 5 min later. The fluid was centrifuged at 300×*g* for 10 min, and the cell pellet and the sterile-filtered supernatant were stored at − 80 °C.

### Biopsy samples

Two biopsies were taken from the vaginal wall of each patient with 3-mm forceps at the 3 and 9 o’clock positions approximately 3–4 cm from the vaginal introitus. One biopsy was fixed in 4% paraformaldehyde at 4 °C overnight, transferred to 70% ethanol and processed for immunohistochemical analysis; a second biopsy was placed in RNA*later* RNA Stabilization Reagent (Qiagen) and subjected to RNA isolation and gene expression analysis.

### Immunofluorescence staining of sections

Biopsies collected for immunohistochemical analysis were paraffin-embedded and cut at 4 μm. Sections were deparaffinized in xylene or Neo-Clear, rehydrated in an ethanol gradient and washed in water and phosphate-buffered saline (PBS). Heat-mediated antigen retrieval was performed in citrate buffer (10 mM, pH 6). Sections were blocked for 30 min with Image-iT FX Signal Enhancer (Life Technologies) and for 60 min with 10% serum from the species that the secondary antibody was raised in. Overnight incubation with primary antibodies was carried out at 4 °C. Primary antibodies used were rabbit anti-HBD1 (1:100, Santa Cruz Biotechnology), goat anti-HBD2 (1:50, R&D Systems), rabbit anti-HBD3 (1:100, Santa Cruz Biotechnology), rabbit anti-psoriasin (1:800, Abcam), and rabbit anti-RNase 7 (1:500, Novus Biologicals). Sections were then incubated with Alexa Fluor-conjugated secondary antibodies (Life Technologies) for 30–60 min at room temperature and mounted in ProlongGold Antifade mounting medium with DAPI (Life Technologies). Images were acquired on a Leica SP5 confocal microscope using a ×40 objective.

### Total RNA extraction and RT real-time PCR

Total RNA extraction from vaginal biopsies and cultured cells was performed using the RNeasy Mini kit (Qiagen). Tissue pieces were lysed in RLT lysis buffer and homogenized with mortar and pestle and then passed through a QIAshredder. Cells were lysed in lysis buffer; samples were further processed according to the manufacturer’s protocol. Reverse transcription with up to 1 μg of RNA was carried out using the DyNAmo cDNA Synthesis Kit (Finnzymes) or the High Capacity cDNA Reverse Transcription Kit (Applied Biosystems) according to the manufacturers’ instructions. Gene expression was analyzed with TaqMan Gene Expression Assays for HBD1 (*DEFB1*, Hs00608345_m1), HBD2 (*DEFB4A*, Hs00175474_m1) and HBD3 (*DEFB103A*, Hs00218678_m1), psoriasin (*S100A7*, Hs00161488_m1), human cathelicidin LL-37/hCAP-18 (*CAMP*, Hs00189038_m1), IL-1β (*IL1B*, Hs01555410_m1), IL-6 (*IL6*, Hs00985639_m1), and IL-8 (*IL8*, Hs00174103_m1). Expression of RNase 7 was examined using a SYBR Green-based assay (Qiagen) with previously described primers [15] and conditions [16]. GAPDH and 18S rRNA were used as internal controls to calculate relative gene expression.

### Western blot detection of psoriasin

For detection of psoriasin in cell pellets, cells were lysed in an equal volume of Triton X-100 (1% in PBS, with protease inhibitors). The lysate was cleared by centrifugation and the protein concentration was determined by BCA assay (Thermo Scientific). Equal amounts of protein were heated in twofold tricine sample buffer (Bio-Rad) at 95 °C for 5 min and subjected to polyacrylamide-gel-electrophoresis using 10–20% tris-tricine gels (Bio-Rad), and the separated samples were transferred to PVDF membranes (Invitrogen). After transfer, membranes were blocked with milk-TBST and incubated with a polyclonal rabbit anti-psoriasin (1:800 in milk-TBST) antibody for 1 h at room temperature at 4 °C. Incubation with an anti-rabbit HRP-conjugated secondary antibody was carried out at room temperature for 60 min. Signals were detected using SuperSignal West Pico Chemiluminescent reagents (Thermo Scientific).

### Enzyme-linked immunosorbent assays (ELISA)

Vaginal lavage samples were analyzed using ELISA kits for human β-defensins (HBD) 1, 2, and 3 (Alpha Diagnostic International); the human cathelicidin LL-37 (Hycult Biotech); human ribonuclease (RNase) 7 (Icosagen); and IL-8 (R&D Systems) according to the manufacturer’s instructions. Samples were tested in appropriate dilutions to fall into the concentration range covered by the internal standards. For HBD3, samples with signals above the limit of the detection system (2000 pg/ml) were assigned a value of 3000 pg/ml to allow statistical evaluation.

### Chemicals and reagents

Psoriasin was purified from human skin [17], dissolved in 0.01% acetic acid to a concentration of 1 mg/ml, and stored at − 20 °C. A vaginal fluid simulant (VFS) was used as medium for all reactions to mimic the vaginal milieu. This previously described formulation resembles the vaginal material with respect to pH, osmolarity, and chemical composition [18]; organic acids are represented by lactic and acetic acid and proteins by albumin. All mono- and polysaccharides were purchased from Sigma; working dilutions were prepared freshly in VFS.

### *Candida albicans* adhesion assays

The adhesive capacity of *C. albicans* isolates was determined in a microtiter plate assay, using VFS as medium or vaginal lavage. *C. albicans* adhesion was measured according to a previously described protocol with modifications [19]. *C. albicans* yeast cells from the logarithmic growth phase were collected by centrifugation, washed twice in PBS, the pellet was suspended in VFS, and the density was adjusted spectrophotometrically. A 100-μl volume containing approximately 1 × 10^6^ colony-forming units (CFU) was added to wells on 96-well sterile, non-treated, flat-bottomed microtiter plates (Corning) and incubated for 30 min at 37 °C with low agitation (100 rpm). Thereafter, wells were washed three times with PBS to remove non-adherent cells, and the number of adherent cells was quantified by metabolic activity using an XTT assay. To evaluate the influence of psoriasin on *C. albicans* adhesion, psoriasin (0.001–1 μM or vector) was added to duplicate wells. In selected experiments, psoriasin (1 μM or vector) was pre-incubated with polysaccharides β-(1,3)-d-glucan from baker’s yeast, d-mannan from baker’s yeast, and chitin from crab shells (1 mg/ml in VFS, all from Sigma) for 30 min at 4 °C prior to the addition of *C. albicans*. To evaluate the effect of vaginal lavage on adhesion, VFS was replaced by vaginal lavage fluid from patients and control participants. The lavage contributed to 40% of the final reaction volume. In some experiments, lavage samples were treated with polysaccharides in the same manner as described for psoriasin.

### Psoriasin-*Candida* binding assays

A pull-down assay was adapted from a previously described protocol [19]. To demonstrate binding between *C. albicans* cells and psoriasin, approximately 1.5 × 10^7^ cells were incubated with 10 μg psoriasin in VFS (final volume 250 μl) and incubated for 30 min at 4 °C with agitation; alternatively, 250 μl of vaginal lavage was used. Cells were collected by centrifugation and washed twice with VFS. Binding of psoriasin to polysaccharides was tested in a similar manner; because of the solubility of mannan, mannan-agarose was used. β-(1,3)-d-Glucan from baker’s yeast, chitin from crab shells (0.25 mg/ml), or mannan-agarose (30 μl) were mixed with 10 μg psoriasin in VFS (final volume 750 μl) and incubated overnight at 4 °C with agitation; samples without any polysaccharide or psoriasin were kept alongside for control purposes. Polysaccharides were then pelleted by centrifugation and washed twice with VFS. The final pellets were heated in tricine sample buffer (Bio-Rad) at 95 °C for 5 min and Western blotting was performed as described above.

### Cell culture

The vaginal epithelial cell line AO was kindly provided by David J. Klumpp, Northwestern University, Chicago, IL, and was used as model of the vaginal epithelium. Cells were cultured in EpiLife Medium with 60 μM calcium and supplemented with Human Keratinocyte Growth Supplement (Gibco) in a humidified incubator at 37 °C with 5% CO_2_.

### Cell infection

*C. albicans* from logarithmic growth phase were collected by centrifugation, washed in PBS. Aliquots of 3 × 10^7^ cells were transferred in low-binding 1.5-ml tubes. Cells were collected by centrifugation, and the pellet was suspended in 98 μl VFS with 2 μl psoriasin (1 mg/ml) or vector. The mixture was incubated 30 min on ice, with occasional mixing. Cells were collected by centrifugation at 4 °C, the supernatant was discarded, and the pellet suspended in 200 μl 0.2% paraformaldehyde in PBS. Cells were fixed for 10 min at room temperature, washed once, and suspended in cell culture medium to a final concentration of 10^7^ CFU/ml. From this suspension, 100 μl was added to a well of confluent AO cells containing 900 μl of fresh cell culture medium and incubated for 6 h at 37 °C with 5% CO_2_ in a humidified incubator. The medium was removed, and cells were collected for RNA extraction and gene expression analysis. All conditions were analyzed in duplicate.

### Statistical analysis

All statistical analyses were performed using GraphPad Prism, Version 6. Data obtained from clinical material were analyzed with non-parametric tests; results are presented as individual values with median. For matched comparisons, the Wilcoxon matched-pairs signed rank test or Friedman test was used; comparison between groups was performed by Mann-Whitney *U* test or Kruskal-Wallis test as appropriate. Variations in the number of participants included were due to limited material, and biological outliers were not excluded. Data from other in vitro experiments are presented with mean and standard deviation from at least three independent experiments (*n* ≥ 3). Results were analyzed by unpaired *t* test, one-way ANOVA with Dunnett’s multiple comparison tests, or two-way ANOVA with Bonferroni’s multiple comparison correction, as appropriate. Technical outliers were detected by Grubb’s test and excluded. All tests were performed two-sided, and differences with *P* values of < 0.05 were considered statistically significant.

## Results

### *Candida albicans* induces production of psoriasin during mucosal infection

**Fig. 1:**
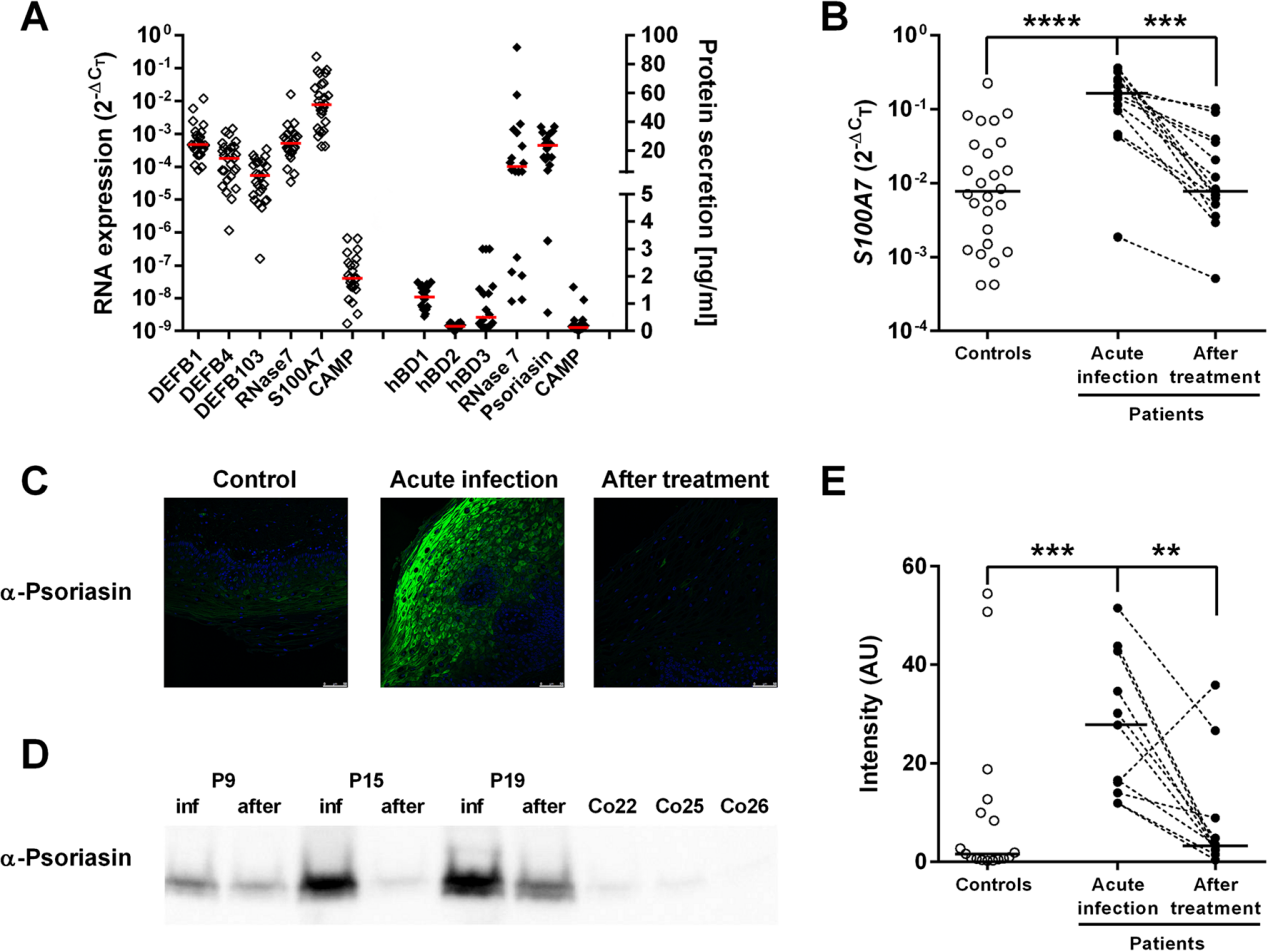
Psoriasin dominates the response to vulvovaginal infection with *Candida albicans*. Vaginal biopsies and lavage samples were collected from healthy women (controls, Co) (**a**–**e**) and patients with recurrent vulvovaginal candidiasis (patients, P) (**b**–**e**). **a** Expression of antimicrobial proteins and peptides in vaginal tissue was determined on the mRNA level by RT-PCR and is expressed in relation to 18S rRNA or GAPDH levels (open diamonds, left axis); secreted proteins and peptides were detected by ELISA (filled diamonds, right axis). **b** S100 mRNA in controls (open circles) and patients during infection and after treatment (filled circles). **c** Immunohistochemistry of tissue sections (psoriasin, green; cell nuclei, blue; ×40 objective). **d** Western blot of lavage samples. **e** Psoriasin-specific bands on Western blots were quantified using ImageJ, and signal intensity is expressed in arbitrary units (AU); one representative blot with patients’ samples during infection (inf) and after treatment (after), and samples from controls are shown. Individual values with median are shown (**a**, **b**, **e)**; analyses were performed with Mann-Whitney test to compared data from controls and patients, and Wilcoxon matched-pairs signed rank test was used to evaluate data from patients during acute infection and after treatment; **P < 0.01, ***P < 0.001, ****P < 0.0001

To describe the expression of epithelial AMPs at baseline and in response to *C. albicans*, mRNA and protein levels of six AMPs were analyzed in vaginal biopsies from healthy women (*n* = 27, Fig. 1a), from patients during acute *C. albicans* vulvovaginitis and from the same patients after successful antifungal treatment (*n* = 16; Fig. 1b and Supplementary Fig. S1). Out of six analyzed AMPs, psoriasin (encoded by the *S1007A* gene) and RNase 7 showed the highest mRNA and protein levels (Fig. 1a). RNase 7 expression was not influenced by infection with *C. albicans* (Supplementary Fig. S1). Psoriasin, on the other hand, was strongly up-regulated during the acute infection episode, both on the mRNA (Fig. 1b) and on the protein levels as shown by immunohistochemistry (Fig. 1c) in vaginal biopsies and by Western blot in the cell pellet from the vaginal lavage samples (Fig. 1d–e). Increased production of psoriasin was primarily detected in the pellet of the vaginal lavage containing desquamated mucosal cells and yeast, and the levels of psoriasin in the lavage supernatant were not significantly affected by infection (Supplementary Fig. S2).

### Psoriasin interacts with β-glucan in the *Candida albicans* cell wall

**Fig. 2:**
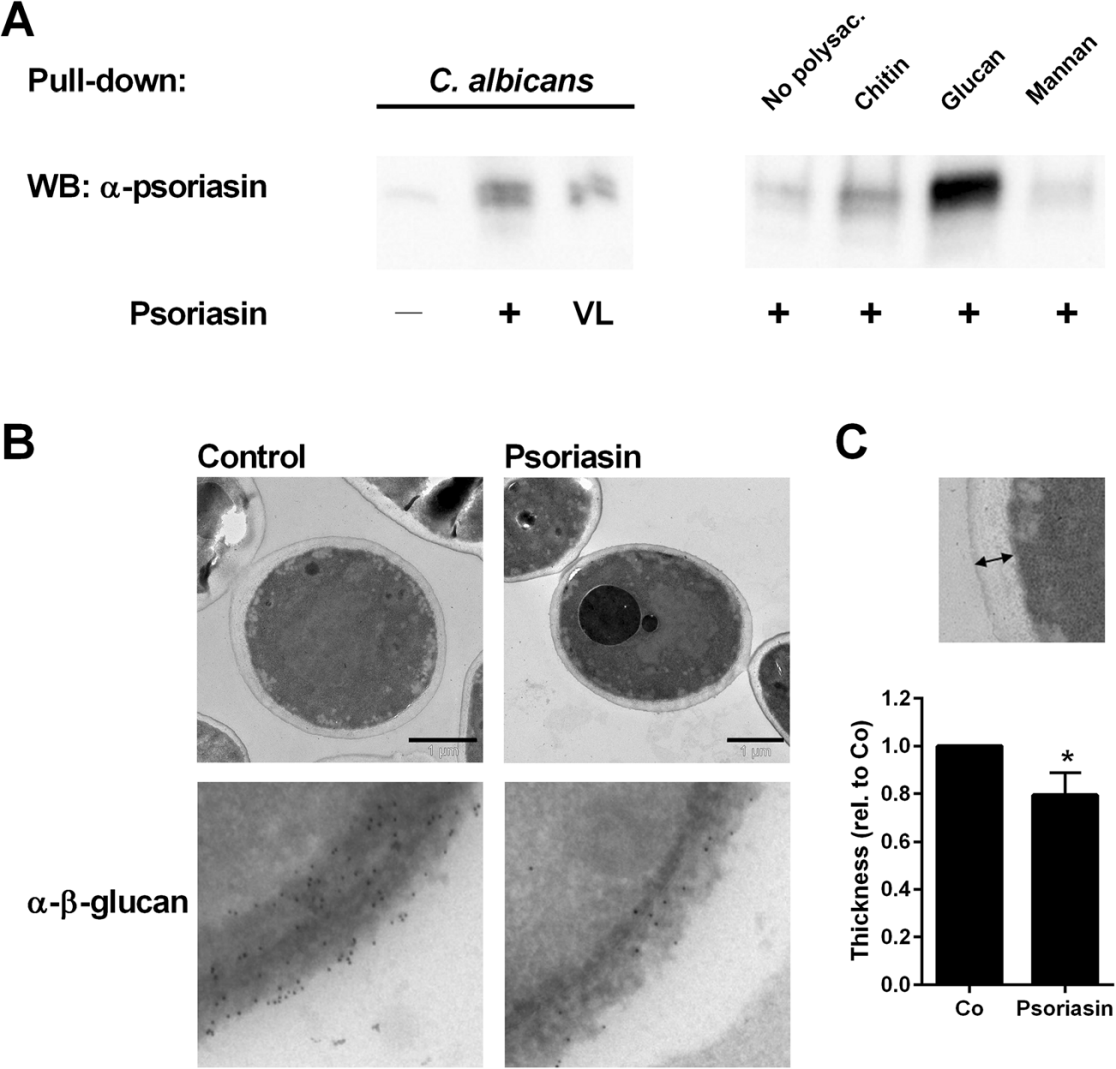
Psoriasin binds to *Candida* β-glucan and reduces its content in the cell wall. **a** *C. albicans* cells or cell wall polysaccharides were incubated with purified psoriasin or vaginal lavage (VL), the pellet was subjected to SDS-PAGE, and membranes were probed for psoriasin. **b** Due to the solubility of mannan, a mannan-agarose conjugate was used. *C. albicans* was incubated with psoriasin or vector (control) for 30 min at 4 °C and investigated by transmission electron microscopy, scale bars 1 μm (upper panel); β-glucan was marked with immunogold-labeled anti-β-glucan antibodies (lower panel, enlarged). **c** The cell wall thickness from non-treated (Co) and psoriasin-exposed cells (Psoriasin) in five to eight images per experiment was measured as exemplified in the close-up; mean results from three independent experiments are presented as mean and standard deviation and compared by unpaired *t* test; *P < 0.05

Since psoriasin produced during acute mucosal infection was associated with the cell pellet and not increased in the supernatant (Fig. 1d and Supplementary Fig. S2), we analyzed a possible interaction between psoriasin and *C. albicans*. We performed a pull-down assay using whole *C. albicans* cells and the polysaccharides β-glucan, chitin and mannan, the most important components of the fungal cell wall. Both purified psoriasin and the psoriasin present in vaginal lavage strongly bound to whole *C. albicans* cells and to β-glucan, while chitin and mannan displayed only weak binding activity to psoriasin (Fig. 2a).

Electron microscopic investigation demonstrated a significant thinning of the cell wall in psoriasin-exposed *C. albicans* (Fig. 2b, c). Moreover, immunogold-labelling revealed diminished content of β-glucan in the affected cells (Fig. 2b).

### Psoriasin inhibits *Candida albicans* adhesion to surfaces

**Fig. 3:**
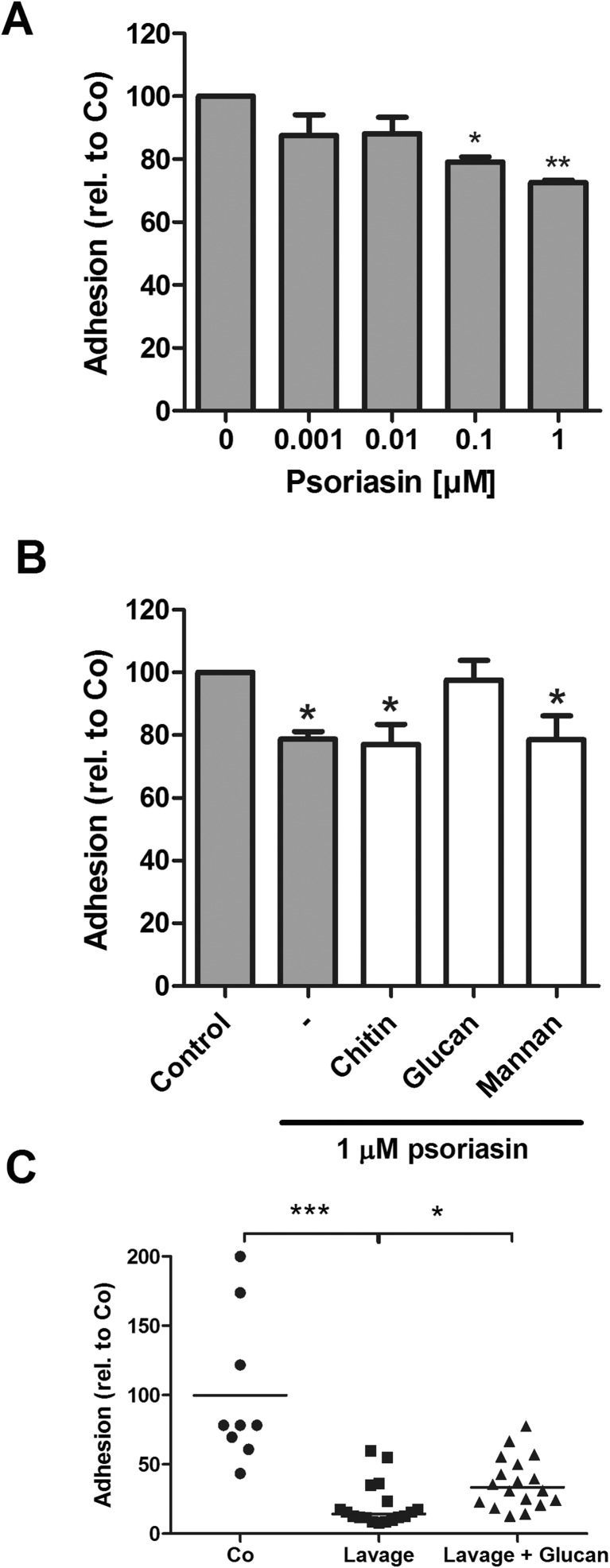
Psoriasin reduces *Candida* adhesion. **a** Adhesion of *C. albicans* in vaginal lavage simulant with different concentrations of psoriasin was quantified by yeast metabolic activity using the XTT assay. **b** The same assay was performed with psoriasin pre-incubated with polysaccharides. Data are expressed in relation to corresponding samples without psoriasin (control, Co = 100) and shown as mean and standard deviation from three to six independent experiments; results were analyzed by one-way ANOVA with Dunnett’s multiple comparison test versus control; *P < 0.05, **P < 0.01. **c** In a similar assay, *Candida* adhesion was measured in vaginal lavage samples from healthy women and from patients with recurrent vulvovaginal candidiasis, and in the presence of glucan (*n* = 18). Individual values with medians are shown, and results were analyzed by Kruskal-Wallis with Dunn’s multiple comparison test; *P < 0.05, ***P < 0.001

To evaluate the effect of psoriasin-mediated changes in the candidal cell wall, we investigated adhesion of *C. albicans* under conditions mimicking the vaginal milieu [18]. Adherence of *C. albicans* cells to polystyrene was reduced by psoriasin in a concentration-dependent manner (Fig. 3a). β-Glucan had the ability to completely neutralize the anti-adhesive properties of psoriasin, which was not the case for chitin or mannan (Fig. 3b).

To confirm the effect also in vaginal lavage, samples from healthy volunteers and from patients with acute *C. albicans* vulvovaginitis were analyzed in a similar adhesion assay. The presence of anti-adhesive activity was confirmed in vaginal lavage fluid and could partially be blocked by β-glucan (Fig. 3c).

### Epithelial immune response to *Candida albicans* is enhanced by psoriasin

**Fig. 4:**
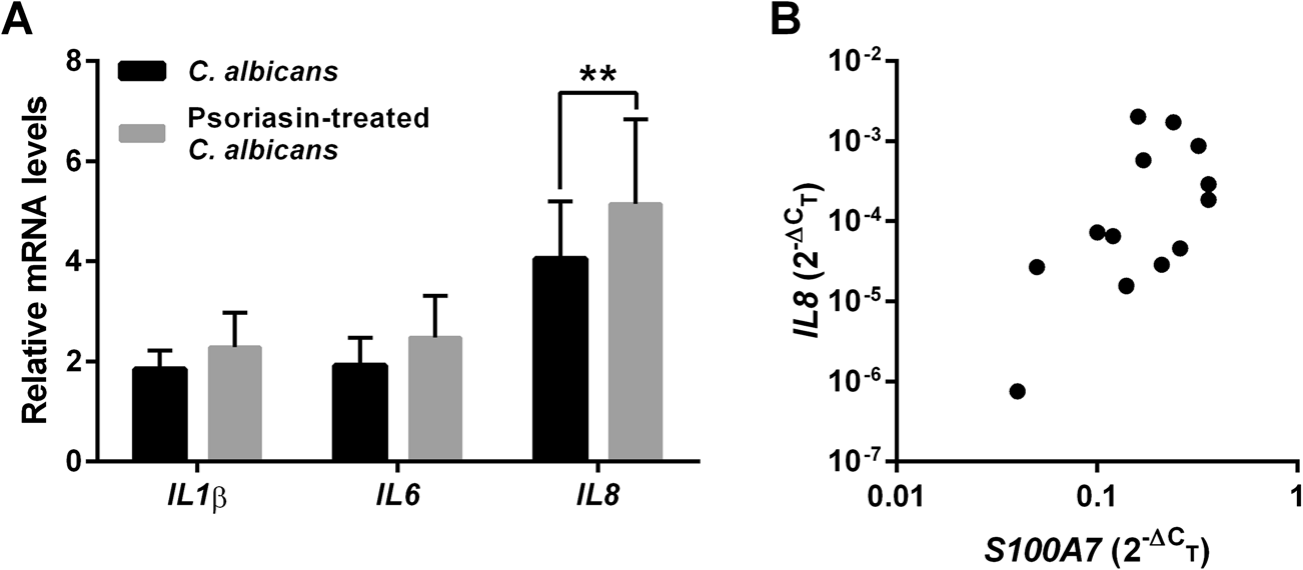
Psoriasin promotes a pro-inflammatory response to *Candida*. **a** Vaginal epithelial cells were infected with *C. albicans* pre-treated with psoriasin or left untreated. After 6 h, induction of cytokine mRNA was determined by RT-PCR. Data from four independent experiments are shown with mean and standard deviation; results were analyzed by two-way ANOVA with Bonferroni’s multiple comparison test. **b** Relative mRNA expression of IL-8 and psoriasin was measured in vaginal tissue from women during an acute infection with *C. albicans*. Individual values (*n* = 14) are shown, correlation between psoriasin mRNA (*S100A7*) and *IL8* was assessed by Spearman’s test, *r* = 0.55, *P* < 0.05

To further analyze the consequences of binding of psoriasin to *C. albicans*, vaginal epithelial cells were infected with psoriasin-treated and untreated fungal cells. Overall, psoriasin treatment of *C. albicans* had an impact on the induction of pro-inflammatory cytokines, with the most pronounced effect on IL-8 (Fig. 4a). Accordingly, *IL8* mRNA levels in patients during acute infection correlated positively with expression levels of psoriasin mRNA (Fig. 4b). However, psoriasin alone did not influence IL-8 expression in epithelial cells in vitro (Supplementary Fig. S3).

## Discussion

We here describe the expression of the antimicrobial protein psoriasin during mucosal infection with *C. albicans* and a novel mechanism of action of this protein. Psoriasin contributes to the inhibition of *C. albicans* adhesion and to the increased immune response to *C. albicans* by mucosal epithelial cells.

*C. albicans* is part of the normal flora on skin and mucosal membranes of humans, but as an opportunistic pathogen can cause both local and systemic infections. Vulvovaginal candidiasis (VVC) is a common local infection in women of reproductive age [20], and some will experience recurrent VVC, with several episodes of acute infections per year [21, 22]. The majority of mucosal as well as skin infections caused by *C. albicans* can be linked to impaired immunity or the presence of foreign bodies like indwelling catheters. In contrast, women suffering from sporadic or recurrent VVC are otherwise healthy without known underlying conditions [23].

Epithelial cells lining the body surfaces are the first to come into contact with invading pathogens and are equipped with AMPs to fend off potential intruders [6]. We analyzed a panel of AMPs potentially involved in mucosal immunity against *C. albicans*. Overall, the pattern resembled a previously reported expression profile [24].

To our surprise, the most abundantly expressed and up-regulated AMP during acute *C. albicans* infection was psoriasin. This S100 protein is a powerful antimicrobial for *E. coli* and to a less degree also some other bacteria [10]. In its cysteine-reduced form, psoriasin is a potent fungicidal for filamentous fungi. However, unexpectedly and for unknown reasons, it is not killing *C. albicans* [10, 12].

In search for alternative actions of psoriasin, we performed a pull-down assay using whole *C. albicans* cells or the major components of the fungal cell wall. Psoriasin strongly bound to whole yeast cells and to β-glucan. Accordingly, *C. albicans* cells were also able to precipitate psoriasin in vaginal lavage samples.

The mode of antibacterial action by psoriasin is still not completely understood. Its activity appears to depend on its zinc-binding motifs [10, 25, 26], which also mediate binding to *E. coli* but not *Staphylococcus aureus* [25]. Membrane integrity, however, was only affected in a Gram-positive species and at low pH [27]. The fungicidal activity of cysteine-reduced psoriasin is based on a selective cellular uptake, possibly mediated by hydrophobin-like proteins, followed by intracellular Zn^2+^-binding and subsequent induction of apoptosis-like cell death in filamentous fungi [12]. It is possible that the absence of hydrophobin-like molecules in *C. albicans* contributes to psoriasin’s inability to kill *C. albicans*.

To study potential consequences of psoriasin-mediated changes of the *C. albicans* cell wall, two major steps in the pathogenesis of Candida infection were investigated, adhesion and induction of a cytokine response [28]. First, our experiments with vaginal lavage from healthy women and from patients with recurrent VVC confirmed the presence of anti-adhesive activity in vivo. This activity could partly be linked to psoriasin as demonstrated by an in vitro assay. Both the anti-adhesive activity present in vivo and the activity demonstrated when using purified psoriasin could partially be blocked by β-glucan. Vaginal lavage is known to have several inhibitory factors, such as proteins from commensal lactobacilli [29]. In the current study, the blocking effect of β-glucan in the lavage samples was not complete, suggesting the presence of additional anti-adhesins in vivo.

Second to adhesion, receptor-mediated contact with the host cell generally induces an immune response. Psoriasin binds to glucans, which are the key fungal pathogen-associated molecular patterns (reviewed in [30]). Binding of β-glucan to the C-type lectin dectin-1 was shown to mediate induction of pro-inflammatory cytokines in intestinal epithelial cells [31] and keratinocytes [32]. Therefore, we expected reduced immune induction in response to the β-glucan-depleted cell wall. Immune response to glucans, however, depends on the size and physical properties of their molecules. Large β-glucan particles induce cytokine production, production of reactive oxygen species, and phagocytosis by neutrophils and macrophages, while soluble β-glucans act as antagonists of these processes [33–35]. In line with these findings, we speculate that treatment of *C. albicans* induces cell wall changes with loss of outer material and increased exposure of β-glucan at the cell surface. The increased IL-8 observed suggests binding to large β-glucan and decreased availability of the soluble ones.

In our in vitro experiments, the IL-8 production by vaginal epithelial cells was increased after pre-treatment of *C. albicans* with psoriasin, possibly by reducing the availability of soluble β-glucan. Thus, inhibition of adhesion and increase of cytokine production, although relatively low, may together promote the mucosal immune protection against *C. albicans*.

Interestingly, although psoriasin was clearly up-regulated during the acute phase of VVC, it could not protect the host from infection. However, it can, however, not be ruled out that patients with a single episode of VVC mount a higher and more effective psoriasin response compared to patients with recurrent infections. Further, gene polymorphism of *DEFB1* has been suggested to be involved in susceptibility to human papillomavirus infection [36]. We therefore speculate that polymorphisms in the psoriasin gene may be associated with recurrent *C. albicans* infections.

A strength of our study is the translational approach including clinical as well as basic science aspects. A limitation is the rather small number of clinical samples from patients with recurrent vulvovaginitis. Although many women were recruited for the first sample, not all fulfilled the strict inclusion criteria. Moreover, clinical samples from women with sporadic VVC would have been an important comparison group. However, patients with sporadic VVC generally use over-the-counter medications and rarely come for gynecology consultations. Furthermore, the observed effect of psoriasin was relatively limited, indicating that other factors may also be involved.

## Conclusion

In conclusion, we describe a novel mechanism of action of psoriasin expressed in the mucosal immunity against *C. albicans*. Our results add to the understanding of the host-pathogen interaction during *Candida* infections and may reveal new targets for antifungal treatment strategies.

## Electronic supplementary material

Figure S1 **Expression of antimicrobials proteins and peptides in the vaginal epithelium.** Vaginal biopsies and lavage samples were collected from healthy women (Controls) and patients with recurrent vulvovaginal candidiasis during acute infection and after antifungal treatment. Expression of antimicrobial proteins and peptides was determined on the mRNA level by RT-PCR and is expressed in relation to 18S rRNA levels (left); secreted proteins and peptides were detected by ELISA in vaginal lavage fluid (middle), and by immunohistochemistry in tissue sections (right; proteins or peptides, red or green; cell nuclei, blue; 40× objective). Individual values (controls, *n* = 18–26; patients, *n* = 11–14) with median are shown; analyses were performed with Mann-Whitney test to compared data from controls and patients, Wilcoxon matched-pairs signed rank test was used to evaluate data from patients during acute infection and after treatment; *P < 0.05, **P < 0.01, ***P < 0.001. (GIF 82 kb)

High resolution image (TIFF 9020 kb)

Figure S2 **Psoriasin levels in vaginal lavage samples.** Vaginal lavage was collected from healthy women (Controls) and patients with recurrent vulvovaginal candidiasis during acute infection and after antifungal treatment. Secreted protein was detected by ELISA. Individual values (controls, *n* = 20; patients, *n* = 11–13) with median are shown. (GIF 12 kb)

High resolution image (TIFF 34 kb)

Figure S3 **Psoriasin does not affect expression of IL-8 in vaginal epithelial cells.** Vaginal epithelial cells were stimulated with purified psoriasin for 24 h. Expression of IL-8 was assessed on mRNA level by RT-PCR (A) and on protein level by ELISA (B). Data from 2 to 6 (mRNA) and 4–6 (protein) independent experiments are shown with mean and standard deviation. (GIF 24 kb)

High resolution image (TIFF 77 kb)

Table S1 (DOCX 13 kb)
