# Supplementary figures and images for: Psoriasin, a novel anti-Candida albicans adhesin
Source: J Mol Med (Berl). 2018 May 7;96(6):537–45. doi: 10.1007/s00109-018-1637-6 (PMC5988767; doi:10.1007/s00109-018-1637-6)

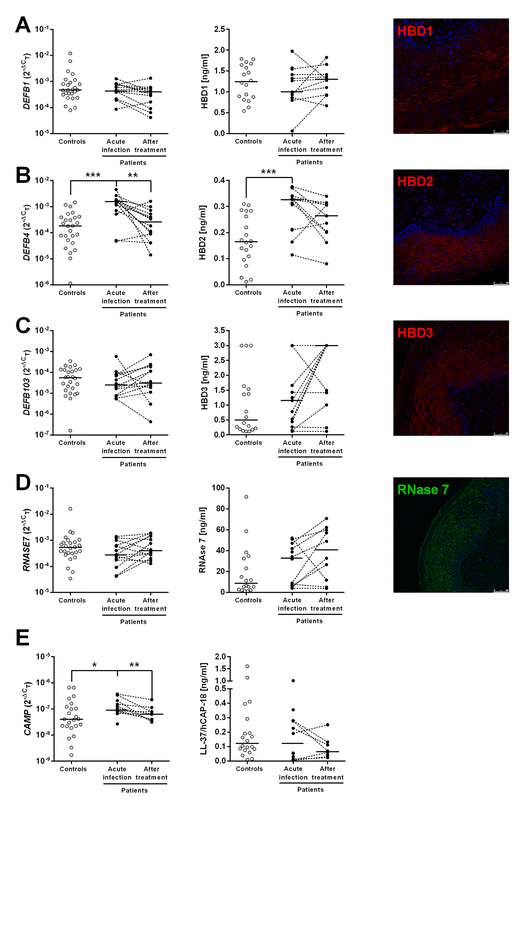

Supplement: Supplementary file 1 — Expression of antimicrobials proteins and peptides in the vaginal epithelium. Vaginal biopsies and lavage samples were collected from healthy women (Controls) and patients with recurrent vulvovaginal candidiasis during acute infection and after antifungal treatment. Expression of antimicrobial proteins and peptides was determined on the mRNA level by RT-PCR and is expressed in relation to 18S rRNA levels (left); secreted proteins and peptides were detected by ELISA in vaginal lavage fluid (middle), and by immunohistochemistry in tissue sections (right; proteins or peptides, red or green; cell nuclei, blue; 40× objective). Individual values (controls, n = 18–26; patients, n = 11–14) with median are shown; analyses were performed with Mann-Whitney test to compared data from controls and patients, Wilcoxon matched-pairs signed rank test was used to evaluate data from patients during acute infection and after treatment; *P < 0.05, **P < 0.01, ***P < 0.001. (GIF 82 kb) [file 109_2018_1637_Fig5_ESM.gif]

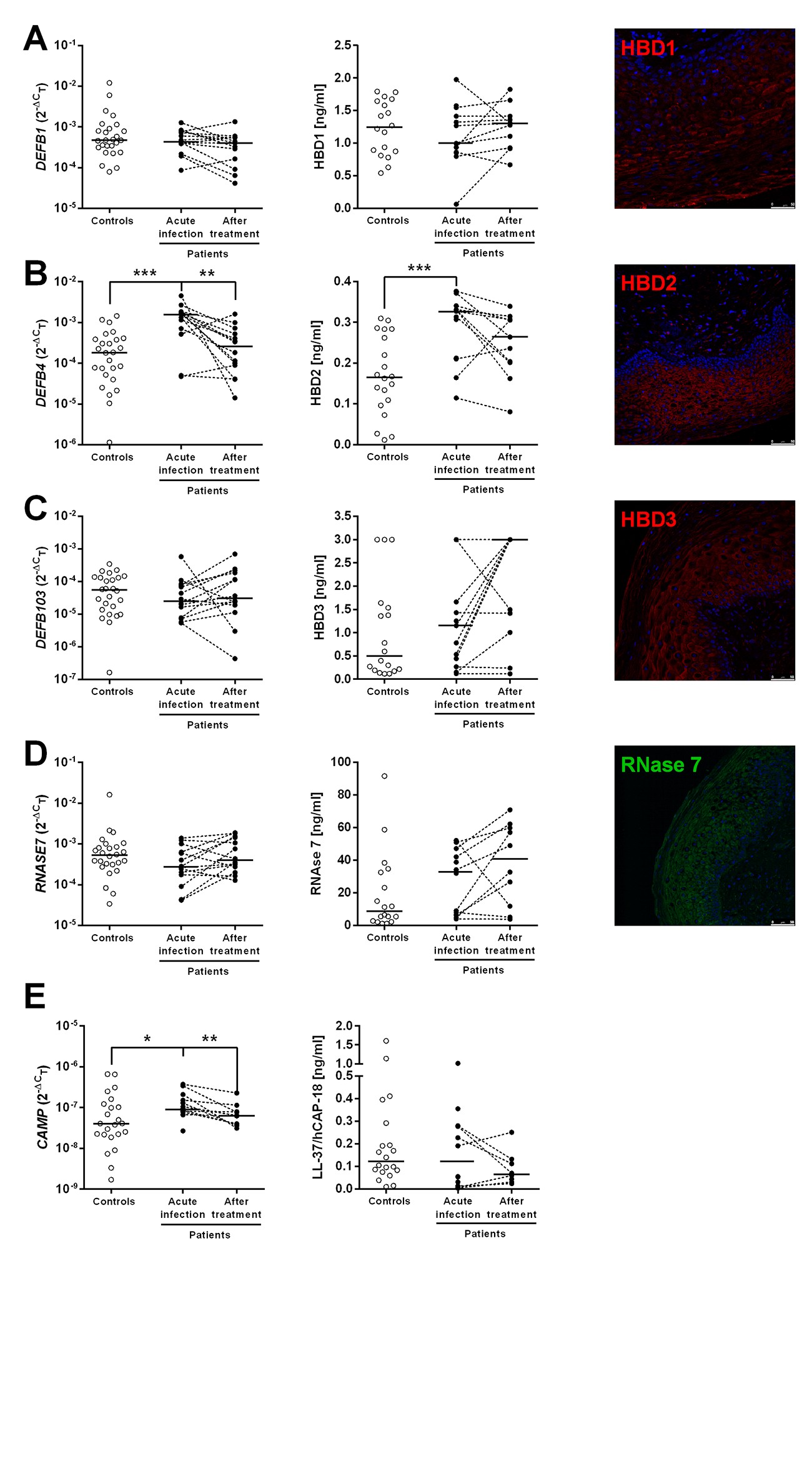

Supplement: Supplementary file 2 — High resolution image (TIFF 9020 kb) [file 109_2018_1637_MOESM1_ESM.tif]

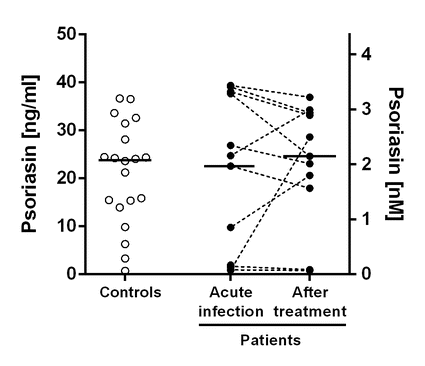

Supplement: Supplementary file 3 — Psoriasin levels in vaginal lavage samples. Vaginal lavage was collected from healthy women (Controls) and patients with recurrent vulvovaginal candidiasis during acute infection and after antifungal treatment. Secreted protein was detected by ELISA. Individual values (controls, n = 20; patients, n = 11–13) with median are shown. (GIF 12 kb) [file 109_2018_1637_Fig6_ESM.gif]

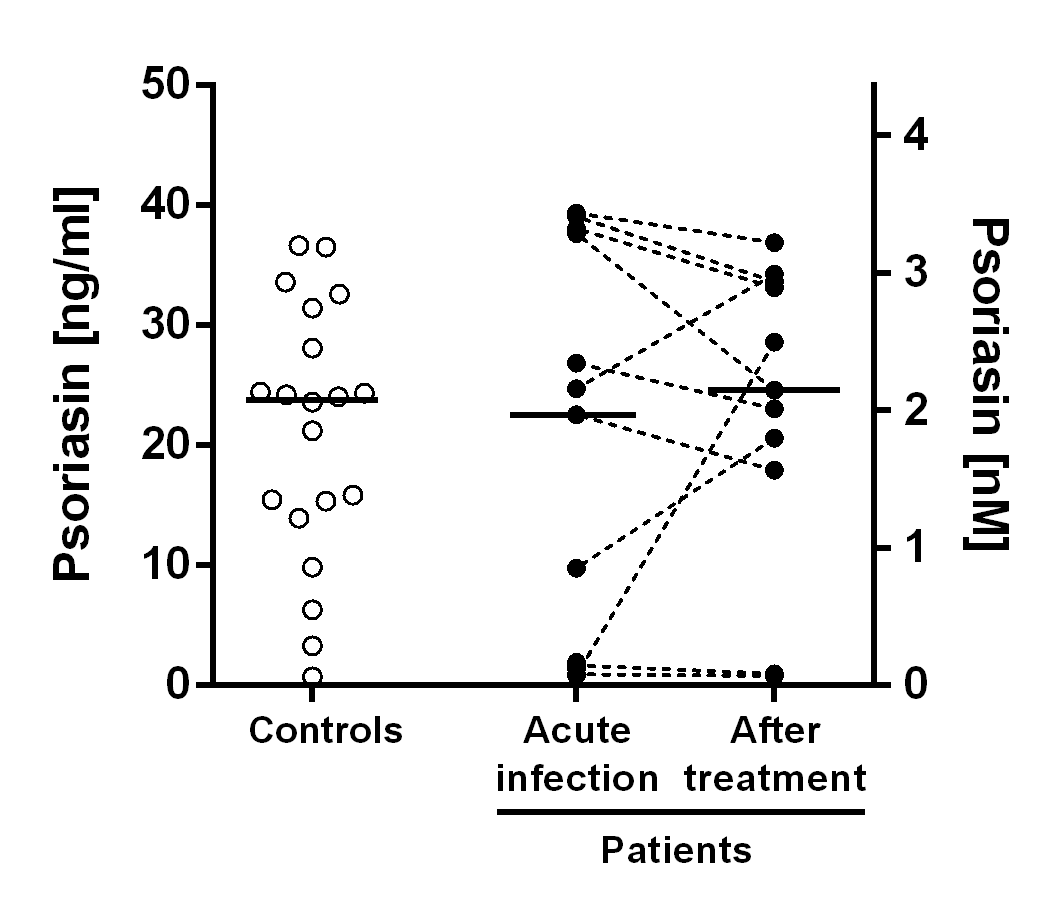

Supplement: Supplementary file 4 — High resolution image (TIFF 34 kb) [file 109_2018_1637_MOESM2_ESM.tif]

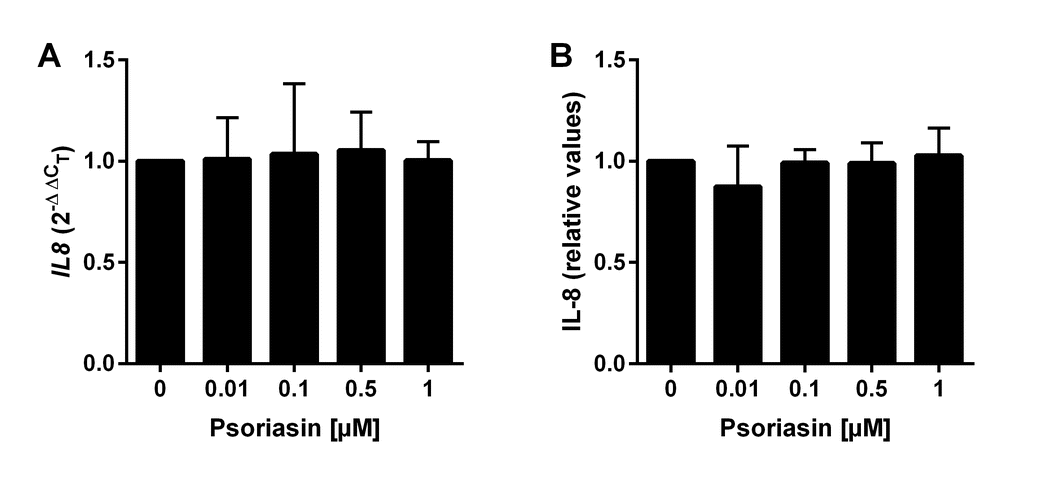

Supplement: Supplementary file 5 — Psoriasin does not affect expression of IL-8 in vaginal epithelial cells. Vaginal epithelial cells were stimulated with purified psoriasin for 24 h. Expression of IL-8 was assessed on mRNA level by RT-PCR (A) and on protein level by ELISA (B). Data from 2 to 6 (mRNA) and 4–6 (protein) independent experiments are shown with mean and standard deviation. (GIF 24 kb) [file 109_2018_1637_Fig7_ESM.gif]

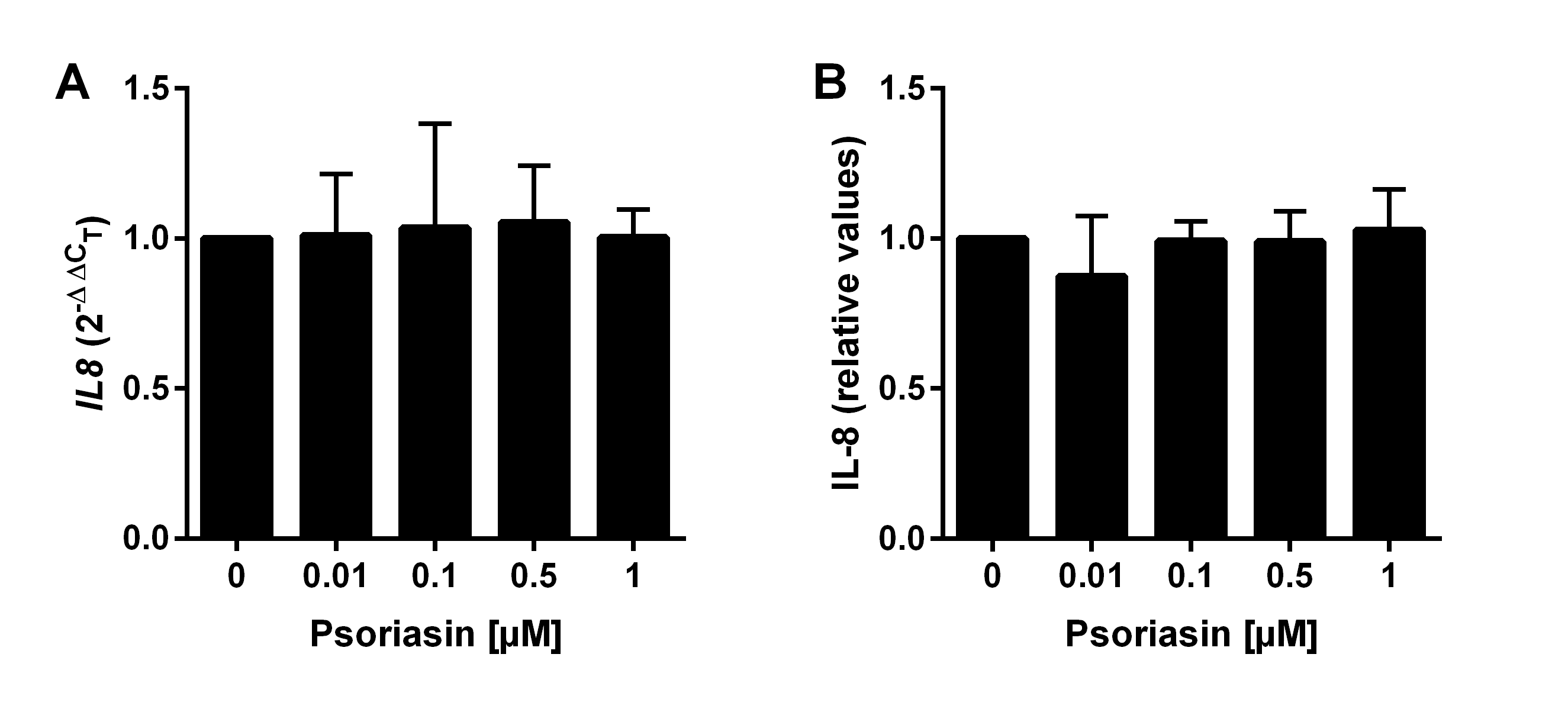

Supplement: Supplementary file 6 — High resolution image (TIFF 77 kb) [file 109_2018_1637_MOESM3_ESM.tif]
