# Supplementary material for: Psoriasin, a novel anti-Candida albicans adhesin
Source: J Mol Med (Berl). 2018 May 7;96(6):537–45. doi: 10.1007/s00109-018-1637-6 (PMC5988767; doi:10.1007/s00109-018-1637-6)
Supplement: Supplementary file 7 — (DOCX 13 kb) [file 109_2018_1637_MOESM4_ESM.docx]

**S1 Table. Use of contraceptives among study participants.**

| **Contraceptives** | **Patients**  ***n*=16 (%)** | **Controls**  ***n*=27 (%)** | ***P*-value*** |
| --- | --- | --- | --- |
| Gestagen method | 4 (25) | 3 (11) | 0.233 |
| Combined method** | 3 (19) | 4 (15) | 0.735 |
| Cupper device | 3 (19) | 4 (15) | 0.735 |
| Condom | 3 (19) | 2 (7) | 0.262 |

* Fisher’s exact test; ** Estrogen/gestagen
